# Introduction of fish and other foods during infancy and risk of asthma in the All Babies In Southeast Sweden cohort study

**DOI:** 10.1007/s00431-018-03312-5

**Published:** 2019-01-07

**Authors:** Sofia Klingberg, Hilde K. Brekke, Johnny Ludvigsson

**Affiliations:** 1 Division of Pediatrics, Department of Clinical and Experimental Medicine, Linköping University, Linköping, Sweden; 2 Department of Public Health and Community Medicine, Section for Epidemiology and Social Medicine (EPSO), Institute of Medicine, Sahlgrenska Academy, University of Gothenburg, P.O. Box 454, 405 30 Gothenburg, Sweden; 3 Department of Nutrition, Institute of Basic Medical Sciences, Faculty of Medicine, University of Oslo, Oslo, Norway; 4 Crown Princess Victoria Children’s Hospital, Linköping University Hospital, Linköping, Sweden

**Keywords:** Introduction of complementary foods, Asthma, Fish, Infant formula, Breastfeeding, Infancy, Cohort

## Abstract

The etiology of asthma includes lifestyle factors. Breastfeeding and introduction of complementary foods have been suggested to affect asthma risk, but the scientific foundation is not solid. Children from the birth cohort All Babies In Southeast Sweden study were included (*n* = 9727). Breastfeeding duration and timing of introduction of infant formula and food were collected prospectively during the first year. Through linkage to the Swedish Patient Register, 948 children were identified with any asthma until age 15–17 years, of which 450 cases were atopic. Breastfeeding duration was not associated to risk of asthma. Introduction of infant formula earlier than at 14 weeks of age was associated with higher risk of non-atopic asthma. Introduction of fish before 43 weeks of age, as compared to later, was associated with a lower risk of asthma, irrespective of atopic classification. Reverse causation was accounted for but did not explain the results.

*Conclusion*: Introduction of infant formula and timing of introduction of fish seem to impact the long-term risk of doctor-diagnosed asthma. Emphasis on the growing body of evidence that early introduction of allergens offers protection against atopic disease should be considered in future recommendations.

**Table:** 

**What is Known:** • *Breastfeeding and introduction of complementary foods might influence the risk of atopic diseases. Recently, a review stated that more studies are needed to clarify the role for asthma development.*	
**What is New:** • *Introduction of infant formula earlier than at 14 weeks of age was associated with a higher risk of developing non-atopic asthma. The risk was pronounced in children introduced to infant formula before 4 weeks of age while being breastfed less than 4 weeks.* • *Early fish introduction, before 43 weeks of age, was associated with a lower risk of asthma, independent of atopic classification of asthma.*

## Introduction

Asthma is a multifactorial disease with an etiology including genetics as well as environmental and lifestyle factors during the prenatal, perinatal, and postnatal periods of life. Duration of breastfeeding and timing of introduction of complementary foods are modifiable lifestyle factors that have been suggested to influence the risk of asthma and atopic disease, but the results are inconclusive. Fish is one of the foods that has been given the most attention, but while some studies have found early fish introduction to independently associate with reduced risk of asthma [3, 9, 11], wheezing [7], allergic rhinitis[9, 16, 18, 23], atopic sensitization [9, 18], eczema [1, 4, 9], and allergy defined as either eczema, asthma, food allergy, or allergic rhinitis [6] in childhood, others have found no such relationships for asthma [4, 16, 18, 23], atopic sensitization [4], and eczema [18]. Timing of introduction of other foods than fish have been less well studied in relation to asthma and atopic disease, but it has been suggested that food diversity, i.e., number of foods introduced during the first year, is protective against atopic disease, and especially so for asthma [21].

Although there seems to be some support of the importance of timing of introduction of complementary foods during infancy in relation to subsequent health outcomes, the scientific foundation for such associations is not solid. A recent review on the evidence of risk and protective factors for childhood asthma stated that more studies are needed to clarify the role of breastfeeding and introduction of foods on the risk of developing asthma [2].

Previously, we showed in a Swedish cohort of infants (ABIS; All Babies In Southeast Sweden), born in 1997 to 1999, that introduction of several common foods and food groups such as potatoes, fruits and berries, vegetables, and porridge took place between 4 and 6 months of age for the vast majority [8]. Introduction of some specific foods such as fish and eggs was however delayed until around 8 or 9 months of age, and a considerable part of the infants were not even introduced to these foods during their first year, which potentially could have impact on the future health of the infants.

Therefore, our aim was to investigate duration of breastfeeding and timing of introduction of complementary foods in relation to the risk of developing asthma during the first 15 to 17 years of life in this Swedish population-based birth cohort, while taking reverse causation into account.

## Methods

### Study population

The ABIS cohort study was initiated in 1997 inviting all babies born in the counties of Östergötland, Småland, Blekinge, and Öland from 1 October 1997 to 1 October 1999. The parents were informed about the ABIS study at the midwifery center during pregnancy and recruited to the study at the maternity ward after delivery [10]. A total of around 21,700 babies were born during this period and 17,055 parents/couples agreed to participate of whom 16,022 completed a baseline study questionnaire after delivery. The cohort has been followed through several re-examinations at different ages of the child [20]. The sample in the present study is based on 9727 children for whom, in addition to the baseline questionnaire, also a food diary covering timing of introduction of complementary foods during the first year as well as duration of breastfeeding was completed (described below). The ABIS study was approved by the Research Ethics Committees of the Faculty of Health Sciences, Linköping University, and the Medical Faculty of Lund University.

### Introduction of complementary foods and duration of breastfeeding

Information on timing of introduction of complementary foods was derived from a food diary which was continuously filled in by the parents over the first year. The food diary has been described in detail elsewhere [8]. In short, the food diary consisted of 25 pre-specified foods and food groups, including breast milk, infant formula (any), follow-on formula or gruel, porridge, milk products, cereals, potatoes, fruits and berries, vegetables, sugar-containing foods, meat, fish, and eggs. The parents registered the date of introduction for each food item or food group. Additionally, cessation of breastfeeding was registered in the food diary. Duration of breastfeeding in the present study includes exclusive, predominant, and complementary breastfeeding [26]. When duration of breastfeeding was not reported in the food diary, information on duration of breastfeeding was obtained from a follow-up questionnaire completed at around 1 year of age.

### Child and parent characteristics

Descriptive information on both children and parents was derived from the baseline questionnaire. Maternal education was divided into three categories: low education (compulsory school or less); intermediate education (practical upper secondary school, theoretical upper secondary school, and folk high school); and high education (university 1–3 years and university degree). Information on maternal smoking during pregnancy and about siblings of the child was dichotomous (yes/no). Mode of delivery was reported as either vaginal birth or caesarean section. Gestational age at delivery was reported in weeks. Atopic heredity was defined by a binary variable created from parental report of food allergy (excluding lactose intolerance), asthma, or other atopic diseases. Information on eczema during the first 6 months was derived from the food diary in which complementary questions about sickness during the first year were reported.

### Case ascertainment

Using personal identification numbers, the children were followed through the National Patient Register until the end of follow-up by end of June 2014. The National Patient Register in Sweden covers both inpatient and outpatient care, but does not include care within the primary care system. Cases of asthma were identified by ICD-10 codes for atopic asthma 45.0, 45.0A, 450B, 450W and 458 and non-atopic 451, 45.1A, 45.1W including unspecified asthma 45.9. Only data on year of diagnosis (i.e., not date) was obtained from the register. If a child was diagnosed with non-atopic or unspecified asthma at one time point and atopic asthma at another time point, the child was categorized as an atopic asthma case irrespective of which diagnosis came first.

### Statistics

Differences in child and parent characteristics between cases and non-cases were assessed by binary logistic regression for categorical variables. Odds ratios (OR) were estimated with 95% confidence intervals (CI). Differences in continuous variables between cases and non-cases were examined by the independent samples *t* test.

We used logistic regression to study the association between duration of breastfeeding and timing of introduction of infant formula and complementary foods in relation to the outcome of asthma. Breastfeeding duration and timing of food introduction were categorized into ascending tertiles, using the highest tertile as the reference category. If a specific food (group) was not introduced during the first year for an individual, time of introduction was set at 52 weeks, thus placing the individual in the reference category. Timing of introduction of infant formula was also categorized into tertiles, but with children never introduced to infant formula in a fourth category, acting as the reference group.

All reported covariates, i.e., atopic heredity, early eczema, duration of breastfeeding, sex of the child, siblings, maternal smoking during pregnancy, maternal education, mode of delivery, and gestational age at birth, were considered to be potential confounders, and were included in the adjusted model. Potential interactions between exposure, i.e., duration of breastfeeding and timing of introduction of infant formula and complementary foods, and the presence of atopic heredity or early eczema, respectively, were explored by including the product term in the respective main model. Interaction between introduction of food and duration of breastfeeding was also investigated. To simplify the interpretation of a potential interaction with duration of breastfeeding, breastfeeding duration was dichotomized into < 6 months and ≥ 6 months. When the interaction term was significant, a stratified analysis was performed. Sensitivity analyses were performed by excluding individuals who were only diagnosed with asthma very early in life, i.e., until the year they turned 2 years.

All statistical analyses were performed in the statistical software package IBM SPSS Statistics Version 20.0. All tests were two-sided. To account for multiple testing, a *p* value of < 0.01 was considered significant.

## Results

During follow-up, 948 out of 9727 children, corresponding to 9.7%, were diagnosed with any asthma. Of these, 450 cases of asthma were ever classified as atopic. The median time of the first asthma diagnosis occurred during the fourth year of life (range 0–15).

Table 1 shows characteristics of the study population by the outcome of any asthma. The odds of developing any asthma was higher in boys, in children delivered by caesarean section, and in children with atopic heredity and in children presenting early eczema (all *p* < 0.001). Protection from being breastfed at least 6 months did not reach significance and maternal smoking during pregnancy and maternal education were both unrelated to the outcome.

**Table 1:** Characteristics of the participating children and their parents for those not developing any asthma and for those developing any asthma, respectively, and corresponding unadjusted odds ratios (OR) (95% confidence intervals (CI)) for the outcome of any asthma

	Participants not developing any asthma (*n* = 8779)	Participants developing any asthma (*n* = 948)	OR (95% CI)	*p* value
Male gender (%) (*n* = 9727)	50.8 (4458)	58.8 (557)	1.38 (1.21–1.58)	< 0.001
Delivered by caesarean section (%) (*n* = 9607)	11.2 (972)	15.8 (148)	1.48 (1.23–1.79)	< 0.001
Atopic heredity (%) (*n* = 9727)	35.3 (3101)	49.4 (468)	1.79 (1.56–2.04)	< 0.001
Early eczema (%) (*n* = 9727)	6.0 (525)	13.3 (126)	2.41 (1.96–2.97)	< 0.001
Breastfed at least 6 months (*n* = 9647)	76.3 (6648)	73.3 (688)	0.85 (0.73–0.99)	0.04
Maternal smoking during pregnancy (%) (*n* = 9692)	8.8 (769)	9.4 (89)	1.08 (0.86–1.36)	0.52
Maternal education (%) (*n* = 9686)				0.42
Low	6.7 (586)	7.8 (74)	1.19 (0.91–1.56)	
Intermediate	59.6 (5206)	59.1 (558)	1.01 (0.88–1.17)	
High	33.7 (2950)	33.1 (312)	1 (ref)	

Table 2 shows OR (95% CI) for asthma (any/atopic/non-atopic) by duration of breastfeeding and age at introduction of complementary foods. Duration of breastfeeding was not significantly related to development of asthma. Timing of introduction of most foods and food groups was unrelated to the odds of developing asthma. However, infants introduced to infant formula before 14 weeks of age had higher odds of developing any asthma compared to infants never introduced. The odds were specifically higher for non-atopic asthma, but in the full model only significantly higher for children introduced earlier than at 2.5 weeks of age. Introduction of fish before 29 weeks or between 29 weeks and 43 weeks, as compared to introduction after 43 weeks, was associated to a decreased risk of developing asthma, irrespective of atopic classification.

**Table 2:** Associations between duration of breastfeeding and age at introduction of complementary foods in relation to the risk of asthma

	Any asthma OR (95% CI)		Atopic asthma OR (95% CI)		Non-atopic asthma OR (95% CI)	
	Crude	Adjusted^a^	Crude	Adjusted^a^	Crude	Adjusted^a^
Number of cases/total	(948/9727)	913/9286	(450/9229)	424/8797	(498/9277)	489/8862
Breastfeeding duration						
< 30 weeks	1.24 (1.04–1.48)	1.11 (0.78–1.58)	1.26 (0.97–1.64)	1.31 (0.96–1.78)	1.22 (0.97–1.53)	0.85 (0.53–1.37)
≥ 30 to < 39 weeks	1.05 (0.89–1.26)	1.02 (0.82–1.25)	1.30 (1.02–1.67)	1.34 (1.03–1.75)	0.87 (0.69–1.10)	0.76 (0.57–1.00)
≥ 39 weeks	1 (ref)	1 (ref)	1 (ref)	1 (ref)	1 (ref)	1 (ref)
Infant formula						
< 2.5 weeks	1.56 (1.30–1.87)*	1.38 (1.12–1.70)*	1.38 (1.06–1.78)	1.19 (0.89–1.60)	1.74 (1.37–2.21)*	1.57 (1.19–2.06)*
≥ 2.5 to < 14 weeks	1.45 (1.20–1.74)*	1.41 (1.14–1.74)*	1.30 (1.00–1.70)	1.27 (0.95–1.71)	1.59 (1.24–2.04)*	1.52 (1.15–2.01)*
≥ 14 weeks	1.39 (1.15–1.67)*	1.29 (1.06–1.57)	1.41 (1.09–1.83)*	1.26 (0.96–1.66)	1.37 (1.05–1.77)	1.30 (1.00–1.70)
Never	1 (ref)	1 (ref)	1 (ref)	1 (ref)	1 (ref)	
Milk						
< 19 weeks	0.96 (0.81–1.13)	0.99 (0.83–1.18)	0.95 (0.76–1.19)	1.01 (0.79–1.30)	0.96 (0.76–1.21)	0.97 (0.76–1.23)
≥ 19 to < 23 weeks	1.01 (0.86–1.19)	1.07 (0.90–1.27)	0.84 (0.67–1.07)	0.89 (0.69–1.14)	1.18 (0.94–1.47)	1.25 (0.99–1.56)
≥ 23 weeks	1 (ref)	1 (ref)	1 (ref)	1 (ref)	1 (ref)	1 (ref)
Potatoes						
< 18 weeks	1.12 (0.96–1.31)	1.20 (1.02–1.42)	1.07 (0.86–1.33)	1.18 (0.93–1.49)	1.18 (0.95–1.46)	1.23 (0.98–1.54)
≥ 18 to < 22 weeks	1.13 (0.95–1.34)	1.23 (1.03–1.47)	0.98 (0.76–1.25)	1.08 (0.84–1.40)	1.29 (1.02–1.62)	1.38 (1.09–1.74)*
≥ 22 weeks	1 (ref)	1 (ref)	1 (ref)	1 (ref)	1 (ref)	1 (ref)
Vegetables						
< 18 weeks	1.05 (0.89–1.25)	1.13 (0.95–1.36)	0.95 (0.75–1.20)	1.05 (0.82–1.35)	1.16 (0.92–1.46)	1.22 (0.96–1.55)
≥ 18 to < 22 weeks	0.89 (0.76–1.04)	0.95 (0.81–1.12)	0.75 (0.60–0.94)	0.82 (0.65–1.04)	1.04 (0.84–1.29)	1.09 (0.87–1.35)
≥ 22 weeks	1 (ref)	1 (ref)	1 (ref)	1 (ref)	1 (ref)	1 (ref)
Fruits and berries						
< 20 weeks	1.08 (0.92–1.27)	1.14 (0.96–1.35)	1.03 (0.82–1.28)	1.09 (0.86–1.38)	1.13 (0.91–1.41)	1.19 (0.95–1.49)
≥ 20 to < 25 weeks	0.96 (0.81–1.13)	1.02 (0.86–1.21)	0.82 (0.65–1.05)	0.88 (0.69–1.13)	1.09 (0.87–1.37)	1.15 (0.92–1.45)
≥ 25 weeks	1 (ref)	1 (ref)	1 (ref)	1 (ref)	1 (ref)	1 (ref)
Cereals with gluten						
< 20 weeks	1.03 (0.87–1.21)	1.08 (0.91–1.28)	1.01 (0.81–1.27)	1.12 (0.88–1.43)	1.04 (0.83–1.30)	1.06 (0.84–1.33)
≥ 20 to < 25 weeks	1.07 (0.91–1.26)	1.10 (0.93–1.31)	0.93 (0.73–1.17)	0.98 (0.76–1.25)	1.22 (0.98–1.52)	1.23 (0.98–1.53)
≥ 25 weeks	1 (ref)	1 (ref)	1 (ref)	1 (ref)	1 (ref)	1 (ref)
Cereals without gluten						
< 21 weeks	0.89 (0.75–1.05)	0.91 (0.77–1.08)	0.88 (0.70–1.12)	0.94 (0.73–1.20)	0.89 (0.71–1.11)	0.89 (0.71–1.12)
≥ 21 to < 28 weeks	0.90 (0.76–1.05)	0.90 (0.76–1.06)	0.90 (0.72–1.13)	0.90 (0.71–1.14)	0.89 (0.72–1.11)	0.90 (0.72–1.12)
≥ 28 weeks	1 (ref)	1 (ref)	1 (ref)	1 (ref)	1 (ref)	1 (ref)
Meat						
< 22 weeks	1.08 (0.91–1.29)	1.14 (0.95–1.37)	1.00 (0.78–1.29)	1.09 (0.84–1.43)	1.15 (0.92–1.46)	1.19 (0.93–1.51)
≥ 22 to < 27 weeks	0.90 (0.77–1.06)	0.94 (0.80–1.11)	0.89 (0.71–1.12)	0.95 (0.75–1.20)	0.91 (0.73–1.13)	0.94 (0.76–1.18)
≥ 27 weeks	1 (ref)	1 (ref)	1 (ref)	1 (ref)	1 (ref)	1 (ref)
Fish						
< 29 weeks	0.61 (0.52–0.72)*	0.73 (0.61–0.86)*	0.52 (0.41–0.66)*	0.67 (0.52–0.87)*	0.71 (0.57–0.88)*	0.77 (0.61–0.97)
≥ 29 to < 43 weeks	0.58 (0.50–0.69)*	0.67 (0.57–0.79)*	0.52 (0.42–0.66)*	0.64 (0.50–0.81)*	0.65 (0.52–0.81)*	0.70 (0.56–0.88)*
≥ 43 weeks	1 (ref)	1 (ref)	1 (ref)	1 (ref)	1 (ref)	1 (ref)
Egg						
< 40 weeks	0.74 (0.63–0.87)*	0.87 (0.73–1.02)	0.64 (0.51–0.81)*	0.80 (0.63–1.02)	0.84 (0.68–1.04)	0.92 (0.74–1.15)
≥ 40 to < 52 weeks	0.81 (0.69–0.96)	0.88 (0.74–1.04)	0.73 (0.58–0.93)	0.82 (0.64–1.05)	0.90 (0.72–1.12)	0.93 (0.74–1.17)
≥ 52 weeks	1 (ref)	1 (ref)	1 (ref)	1 (ref)	1 (ref)	1 (ref)

^a^Adjusted for early eczema (yes/no), atopic heredity (yes/no), duration of breastfeeding (days, continuous), sex of child (binary), siblings (yes/no), maternal smoking during pregnancy (yes/no), maternal education (low/intermediate/high), mode of delivery (vaginal/caesarean section), and gestational age at birth (weeks)

*p < 0.01

No significant interactions were found. However, since cessation of breastfeeding and timing of introduction of infant formula coincide and both factors may influence the risk of asthma, we created specific categories by combining duration of breastfeeding and timing of infant formula introduction in an attempt to separate the effect of these two factors. Table 3 shows that for non-atopic asthma, early introduction of infant formula seems to increase the risk regardless of if the children were breastfed less than 4 weeks or at least 16 weeks. After adjustments for covariates, the risk was only significantly increased in children being breastfed less than 4 weeks.

**Table 3:** Combined categories of timing of infant formula introduction and breastfeeding duration in relation to risk of asthma

	Any asthma OR (95% CI)		Atopic asthma OR (95% CI)		Non-atopic asthma OR (95% CI)	
	Crude	Adjusted^a^	Crude	Adjusted^a^	Crude	Adjusted^a^
Number of cases/total	(703/7571)	681/7283	(336/7204)	321/6923	(367/7235)	360/6962
Infant formula						
Infant formula introduced < 4 weeks and breastfed< 4 weeks (*n* = 269)	1.47 (1.00–2.17)	1.16 (0.67–2.09)	0.58 (0.25–1.32)	0.24 (0.08–0.75)	2.34 (1.51–3.61)*	2.19 (1.33–3.60)*
Infant formula introduced < 4 weeks and breastfed ≥ 16 weeks (*n* = 1148)	1.51 (1.22–1.85)*	1.35 (1.08–1.68)*	1.45 (1.08–1.94)	1.25 (0.91–1.71)	1.56 (1.18–2.06)*	1.42 (1.06–1.90)
Infant formula introduced ≥ 16 weeks and breastfed ≥ 16 weeks (*n* = 1335)	1.38 (1.13–1.68)*	1.27 (1.02–1.57)	1.41 (1.07–1.86)	1.18 (0.87–1.60)	1.35 (1.02–1.78)	1.31 (0.99–1.74)
Never introduced to infant formula and breastfed ≥ 16 weeks (*n* = 4819)	1 (ref)	1 (ref)	1 (ref)	1 (ref)	1 (ref)	1 (ref)

^a^Adjusted for early eczema (yes/no), atopic heredity (yes/no), duration of breastfeeding (days, continuous), sex of child (binary), siblings (yes/no), maternal smoking during pregnancy (yes/no), maternal education (low/intermediate/high), mode of delivery (vaginal/caesarean section), and gestational age at birth (weeks)

*p < 0.01

Excluding cases diagnosed with any asthma (*n* = 234), atopic asthma (*n* = 45), or non-atopic asthma (*n* = 150), respectively, during the first 2 years did essentially not have any impact on the results (data not shown).

## Discussion

In this study of a Swedish population-based cohort, we showed that early and intermediate introduction of fish, as compared to late introduction, was consistently associated with a decreased risk of asthma, irrespective of atopic classification, and independent of atopic heredity, eczema before 6 months of age, duration of breastfeeding, and other covariates. Our results further suggest that early introduction of infant formula in combination with short duration of breastfeeding increases the risk of non-atopic asthma. Support for our main findings is found in previous studies also showing that early introduction of fish is associated with a lower risk of asthma [3, 9, 11] or asthma-like symptoms [7]. Furthermore, the absence of infant formula feeding has been shown to associate with a lower risk of doctor-diagnosed asthma in the past 12 months in 10-year-old children, without eczema or without atopic heredity, as compared to introduction of infant formula before 2 weeks of age [19]. The high content of omega-3 long-chain polyunsaturated fatty acids has been suggested to be involved in mechanisms behind the protection from fish against atopic disease. A recent meta-analysis including 5 randomized controlled trials did however not find any significant associations between omega-3 supplementation, starting in infancy, and the risk of asthma [15]. This indicates that there are probably other nutrients that are involved in the protection from early fish introduction. Vitamin D and zinc, which are found in substantial amounts in fish, have, for example, been suggested to have impact on wheeze and asthma [17]. In the present study, we were not able to distinguish between different types of fish, and we can therefore not provide evidence for which nutrients that may be involved in the inverse association between timing of fish introduction and risk of asthma. Oral tolerance [25], developed by ingestion of food allergens, could also be part of the explanation to why early fish introduction is associated to a decreased risk of especially atopic asthma. However, the protective pattern of early fish introduction was only slightly weaker for non-atopic asthma, pointing out that other mechanisms are probably involved.

One potential mechanistic link between infant formula feeding and increased risk of asthma may lie in the mechanistic target of rapamycin complex 1 (mTORC1) kinase [14]. mTORC1 regulates cell growth, cell proliferation, and autophagy and is activated by amino acids. At the time of the study, cow’s milk-based infant formula had a higher protein content as compared to human milk, with specifically higher content of the amino acid leucine, which exaggerates mTORC1 signaling, resulting in both disturbed immune cell programming, with direct impact for asthma development, and excessive weight gain [13]. Rapid weight gain in infancy [22] and peak weight velocity [12] has been associated with increased risk of asthma in population-based samples. However, we were not able to discriminate different types of infant formula, but according to the time when our study was performed, the majority was likely given cow’s milk-based infant formula. Even though the protein content in cow’s milk-based infant formulas has been reduced since the late 1990s, the concentration of leucine is still considerably higher in human milk.

There is a considerable risk of reverse causation since children showing early signs of atopic sensitivity or with heredity for such diseases might be introduced to complementary foods later. For example, atopic dermatitis often appears before the onset of other atopic diseases [24]. This could result in false associations implying late introduction to increase the risk of the outcome. In addition to adjustment for a range of possible confounders in all analyses, we considered reverse causation by examining interactions between timing of introduction and two covariates that could contribute to this phenomenon, namely atopic heredity and early eczema. However, no significant interactions were found. Other factors such as early exposure to respiratory syncytial virus and rhinovirus promote asthma development [5] and could potentially have confounded the present analyses if such exposure also associates with the present exposure, i.e., introduction of infant formula and complementary foods. However, since day care is not provided before the age of 1 year in Sweden and because analyses were adjusted for siblings, it is not likely that the associations presented are confounded by early exposure to respiratory viruses.

Timing of introduction was categorized into thirds for all the investigated foods and food groups. Another strategy would have been to use categories derived from recommendations on timing of introduction of complementary foods. Because very few infants were introduced earlier than recommended for most foods and a large part of the infants were introduced late to some foods (i.e., fish and eggs), this strategy was however not considered as appropriate. In post hoc analysis, we did however perform the analysis with exposure categories primarily derived from recommendations (not shown). Although the results pointed in the same direction, several associations lost significance, probably as a result of reduced statistical power.

Strengths of the present study include the prospective design with a large population-based sample. The prospectively collected data minimizes the risk of recall bias that could occur when dietary data is collected retrospectively. However, it must be acknowledged that exposure data was indeed self-reported. Another strength is that cases were defined by doctor diagnosis retrieved from a high-quality register implying that cases were identified with high sensitivity. However, this study is not without its weaknesses and the fact that the register does not include care given within primary care is one. The majority of childhood asthma cases are however most likely treated in pediatric clinics covered by the register, implying that most cases were actually retrieved from the register. Another weakness is that those not reporting introduction during the first 12 months were considered as not being introduced, but could potentially also be because they forgot to report introduction of a specific food.

## Conclusion

Taken together, our results suggest that introduction of complementary foods, especially introduction of infant formula, and timing of introduction of fish do influence the long-term risk of doctor-diagnosed asthma. Current recommendations on infant nutrition, in Sweden published by the Swedish National Food Agency, stress that infants should be fully breastfed the first 6 months of age starting with tiny tastes of complementary foods, e.g., gluten-containing products, no earlier than at 4 months and without competing with breastfeeding. Although recommendations to avoid introduction of allergens to infants were withdrawn quite a few years ago, emphasis on the growing body of evidence that early introduction of allergens offers protection against atopic disease should be considered in future recommendations. In order to reduce the risk of developing asthma, our results support the following recommendations: do not exclude or delay food groups when introducing complementary foods, do not introduce complementary foods before 4 months of age, and support breastfeeding.

## Abbreviations

ABIS: All Babies In Southeast Sweden
CI: Confidence interval
mTORC1: Mechanistic target of rapamycin complex 1
OR: odds ratio
